# Risk Factors for Acute Kidney Injury in Adult Patients With COVID-19: A Systematic Review and Meta-Analysis

**DOI:** 10.3389/fmed.2021.719472

**Published:** 2021-12-06

**Authors:** Xiaoyue Cai, Guiming Wu, Jie Zhang, Lichuan Yang

**Affiliations:** ^1^Chengdu BOE Hospital, Chengdu, China; ^2^Dazhou Central Hospital, Dazhou, China; ^3^West China School of Medicine, West China Hospital, Sichuan University, Chengdu, China

**Keywords:** risk factor, acute kidney injury, COVID-19, systematic review, meta-analysis

## Abstract

**Background and Objective:** Since December 2019, coronavirus disease 2019 (COVID-19) has spread rapidly around the world. Studies found that the incidence of acute kidney injury (AKI) in COVID-19 patients was more than double the incidence of AKI in non-COVID-19 patients. Some findings confirmed that AKI is a strong independent risk factor for mortality in patients with COVID-19 and is associated with a three-fold increase in the odds of in-hospital mortality. However, little information is available about AKI in COVID-19 patients. This study aimed to analyse the risk factors for AKI in adult patients with COVID-19.

**Methods:** A systematic literature search was conducted in PubMed, EMBASE, Web of Science, the Cochrane Library, CNKI, VIP and WanFang Data from 1 December 2019 to 30 January 2021. We extracted data from eligible studies to compare the effects of age, sex, chronic diseases and potential risk factors for AKI on the prognosis of adult patients with COVID-19.

**Results:** In total, 38 studies with 42,779 patients were included in this analysis. The meta-analysis showed that male sex (OR = 1.37), older age (MD = 5.63), smoking (OR = 1.23), obesity (OR = 1.12), hypertension (OR=1.85), diabetes (OR=1.71), pneumopathy (OR = 1.36), cardiovascular disease (OR = 1.98), cancer (OR = 1.26), chronic kidney disease (CKD) (OR = 4.56), mechanical ventilation (OR = 8.61) and the use of vasopressors (OR = 8.33) were significant risk factors for AKI (*P* < 0.05).

**Conclusions:** AKI is a common and serious complication of COVID-19. Overall, male sex, age, smoking, obesity, hypertension, diabetes, pneumopathy, cardiovascular disease, cancer, CKD, mechanical ventilation and the use of vasopressors were independent risk factors for AKI in adult patients with COVID-19. Clinicians need to be aware of these risk factors to reduce the incidence of AKI.

**System Review Registration:** PROSPERO, identifier [CRD42021282233].

## Introduction

Since December 2019, a novel coronavirus called severe acute respiratory syndrome coronavirus 2 (SARS-CoV-2) has caused an international outbreak of respiratory illness described as coronavirus disease 2019 (COVID-19). As of 30 January 2021, approximately 102,638,000 cases have been confirmed worldwide, and 2,216,546 deaths have occurred.

The main manifestation of COVID-19 is acute respiratory infection, and the renal, cardiovascular, digestive, blood and nervous systems may be simultaneously involved (1, 2). Acute kidney injury (AKI) is a common condition in critically ill patients, particularly in those with serious infections, and has been found to be associated with substantial morbidity and mortality (3). Current evidence suggests four broad mechanisms of renal injury: hypovolaemia, acute respiratory distress syndrome (ARDS), cytokine storms and direct viral invasion, as seen on renal findings during autopsies (4). Most patients have significant insensible fluid loss due to high-grade pyrexia and tachypnoea on presentation (5). These patients are particularly prone to developing pre-renal AKI. Impaired gaseous exchange with hypercapnia leads to a reduction in the renal vasodilatory response and renal blood flow, with altered diuresis and increased oxygen utilisation in the proximal tubule. Severe hypoxemia also causes a reduction in renal blood flow with possible activation of the hypoxia-inducible factor system, influencing lung and kidney outcomes (6). Observational data from a subgroup of patients with COVID-19 suggested the development of features consistent with cytokine storm syndrome triggered by SARS-CoV-2 and characterised by high serum ferritin, D-dimer, lactate dehydrogenase, and IL-6 levels; cytopenia; ARDS; acute cardiac injury; abnormal liver function test results; and coagulation abnormalities (7). In addition, this hyperinflammatory state could cause AKI.

Recently, several clinical studies have demonstrated that AKI is one of the most common complications in patients with COVID-19, and several studies have shown that the mortality rate of COVID-19 patients with AKI is incredibly high, ranging from 8 to 23% (8). It has also been reported that the incidence rate of AKI in COVID-19 patients ranges from 0.5 to 29% depending on disease severity. The AKI incidence rate was found to be 0.1-2% in patients with for mild cases, 3–3.2% in those with severe cases, and up to 8.3–29% in critically ill patients who needed to be admitted to the ICU (9). AKI prolongs the length of hospital stay, increases the cost of hospitalisation, and even increases the risk of death. Therefore, if we can identify the risk factors for AKI in patients with COVID-19 early and initiate preventive measures, we could improve the prognosis of patients. In this article, we performed a systematic review and meta-analysis to explore the characteristics of high-risk groups to provide reliable evidence that can be used to guide clinical practice.

## Methods

### Inclusion and Exclusion Criteria

Make inclusion and exclusion criteria according to PECOS principles.

Inclusion criteria:

Population = patients age > 16 years old with confirmed Covid-19 through any detection methods.

Exposure = patients with one of the following conditions: smoking, obesity, hypertension, diabetes, pneumopathy, cardiovascular disease, cancer, chronic kidney disease (CKD), mechanical ventilation and the use of vasopressors.

Comparison/Control = patients without these following conditions: smoking, obesity, hypertension, diabetes, pneumopathy, cardiovascular disease, cancer, chronic kidney disease (CKD), mechanical ventilation, and the use of vasopressors.

Outcomes = the prevalence of acute kidney injury (AKI) in both group. AKI was diagnosed by using 2012 Kidney Disease Improving Global Outcomes (KDIGO) guidelines.

Study Design = The study types were randomised controlled trials or non-randomised studies (horizontal cross-sectional studies, case-control studies, and cohort studies).

Exclusion criteria: Studies were excluded if the subjects were not representative of the general population, the diagnostic criteria for COVID-19 were not defined, the diagnostic criteria for AKI were not defined, and there was no control group. Studies that were unpublished or duplicate reports and those with incomplete information or logical errors were excluded. Reviews, case reports, conference abstracts, animal studies and basic research were also excluded.

### Search Strategy

A systematic literature search was conducted in PubMed, EMBASE, Web of Science, Cochrane, CNKI, VIP, and WanFang Data from 1 December 2019 to 30 January 2021. The following Medical Subject Heading terms and free words were used, as shown in Table 1: “2019-nCoV” or “SARS-CoV-2” or “COVID-19” or “coronavirus disease 2019” and “acute kidney injury” or “acute kidney failure” or “acute renal failure” or “acute renal injury” or “AKI” and “risk factor” or “influence factor.”

**Table 1:** Search strategy.

**Databases**	**PubMed, EMBASE, Web of Science, Cochrane, CNKI, VIP, and WanFang Data**
Data	1 December 2019 to 30 January 2021
#1	“2019-nCoV” or “SARS-CoV-2” or “COVID-19” or “coronavirus disease 2019”
#2	“acute kidney injury” or “acute kidney failure” or “acute renal failure” or “acute renal injury” or “AKI”
#3	“risk factor” or “influence factor”
Search	#1 and #2 and #3

### Study Selection and Data Collection

Two investigators independently scanned all the titles and abstracts to identify studies that met the inclusion criteria, and they extracted the relevant data from those studies. Any discrepancies between the reviewers were resolved by discussion with a third reviewer. The titles, abstracts and full texts of all initially identified documents were assessed, and those reporting AKI in COVID-19 patients were included in this analysis. The reference lists of all identified studies were also analysed to identify additional eligible studies. Data were collected and entered into a spreadsheet. We extracted the following variables: author, study period, location, and patient age, sex and clinical characteristics. The Newcastle-Ottawa Scale (NOS) was used as a bias assessment tool for cohort studies and case-control studies, and a score ≥ 7 indicated good quality. We performed sensitivity analyses to identify which studies caused the observed heterogeneity. The exclusion of each study one at a time did not significantly alter the results for each factor or the heterogeneity.

### Statistical Analysis

The meta-analysis was performed using RevMan 5.4. Mantel-Haenszel was used for statistical method of dichotomous, and Inverse Variance was used for continuous. The mean differences (MDs) and 95% confidence intervals (CIs) were calculated for continuous data. The odds ratios (ORs) and 95% CIs were calculated for dichotomous data. The I^2^ statistic was used to assess the statistical heterogeneity. If I^2^ ≤ 50%, there is little heterogeneity. Otherwise, it can be considered that there is large heterogeneity. Since the included studies were not RCT study, random-effects model was used for analyses. Potential study bias was assessed using funnel plots.

## Results

### Search Results and Characteristics of the Included Studies

**Figure 1:**
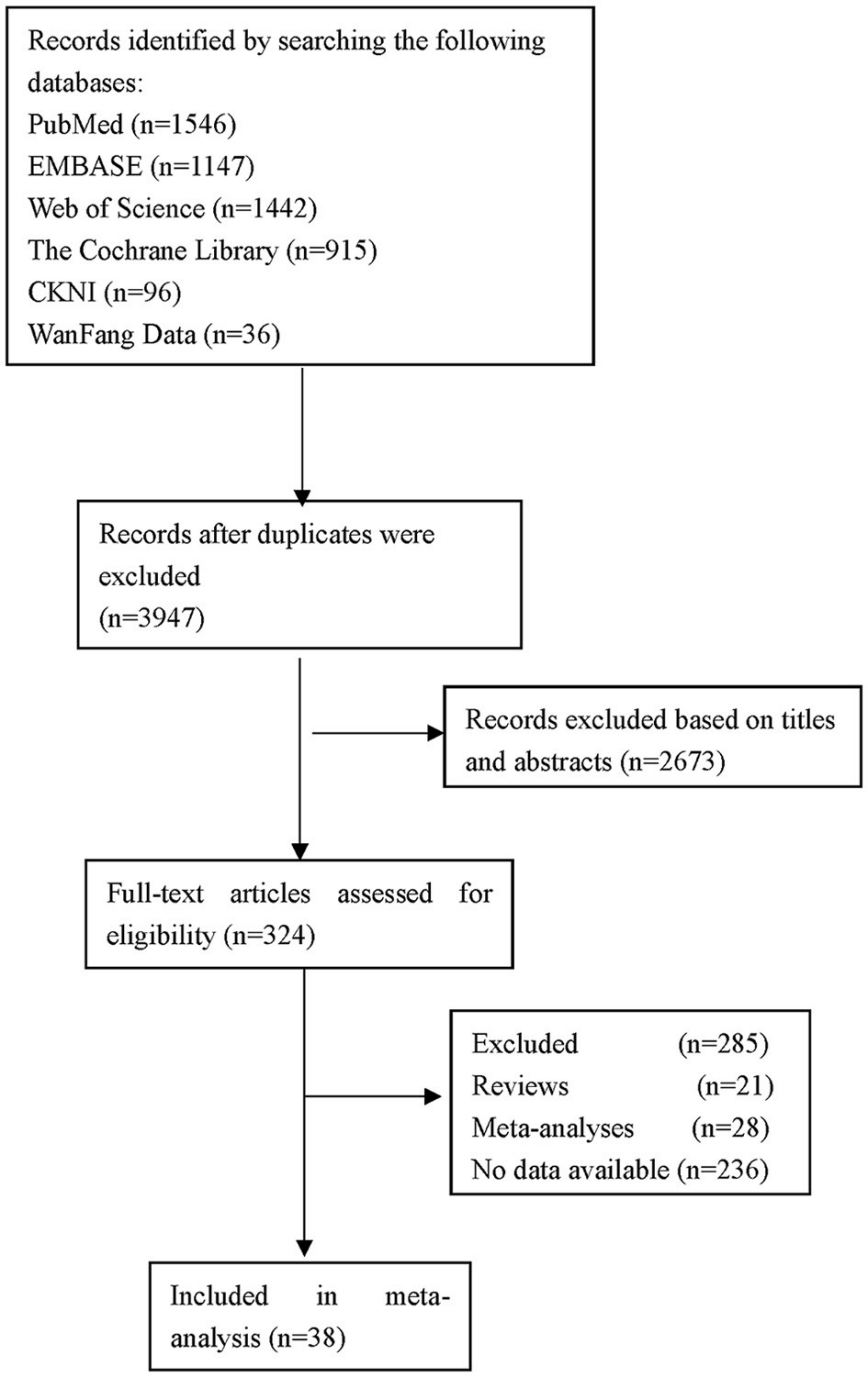
Flow diagram of the selection of studies.

The flow of studies through the analysis is presented in Figure 1. A total of 38 eligible studies involving 42,779 patients were ultimately enrolled in our study, including 18 studies from Asia, 8 studies from Europe and 12 studies from America. The characteristics of the included studies and bias risk assessment results are described in Table 2. About 33 studies scored ≥ 7 by using NOS tool. This indicated that most studies are good quality and the risk of bias assessment is low.

**Table 2:** Characteristics of the 39 studies included in the meta-analysis.

**References**	**Country**	**Type of study**	**NOS score**	**AKI**	**Non-AKI**	**Age**	**Male (*n*, %)**	**CKD (%)**	**Hypertension (%)**	**Diabetes (%)**	**COPD (%)**	**CVD (%)**	**Cancer (%)**
Xu et al. (10)	China	Case-control	5	18	44	62.9 ± 15.3	39 (62.9)	2 (3.2)	26 (41.9)	9 (14.5)	5 (8.1)	11 (17.7)	3 (4.8)
Doher et al. (11)	Brazil	Cohort	8	101	100	64 (52,80)	123 (61.2)	NA	98 (48.8)	64 (31.8)	19 (9.5)	16 (8.0)	NA
Kolhe et al. (12)	England	Cohort	7	304	857	NA	657 (56.6)	224 (19.3)	NA	255 (22.0)	311 (26.8)	117 (10.1)	102 (8.8)
Li et al. (13)	China	Cohort	7	48	59	70	69 (64.5)	5 (4.7)	73 (68.2)	22 (20.6)	23 (21.5)	33 (30.8)	NA
Bowe et al. (14)	United States	Cohort	8	1,655	3,561	70 (61,76)	4,908 (94)	NA	3,985 (76)	2,537 (49)	1,302 (25)	1,588 (30)	799 (15)
Zahid et al. (15)	United States	Cohort	8	128	341	66 (55,75)	268 (57.1)	50 (10.7)	323 (68.9)	219 (46.7)	34 (7.3)	74 (15.8)	31 (6.6)
Taher et al. (16)	Bahrain	Cohort	7	29	44	54.3 ± 13.5	44 (60.3)	6 (8.2)	31 (42.5)	33 (45.2)	NA	9 (12.3)	5 (6.8)
Chan et al. (17)	United States	Case-control	7	1,406	1,829	66.5 (55.6,77.8)	1,868(57.7)	323 (10)	1,193 (36.9)	800 (24.7)	NA	281 (8.7)	NA
Hirsch et al. (18)	United States	Cohort	8	1,993	3,456	64.0 (52.0, 75.0)	3,317 (60.9)	NA	3,037 (55.7)	1,797 (33.0)	296 (5.4)	600 (11.0)	327 (6.0)
Joseph et al. (19)	France	Cohort	7	81	19	59 (53,67)	70 (70.0)	29 (29.0)	56 (56.0)	30 (30.0)	2 (2.0)	15 (15)	NA
Cui et al. (20)	China	Case-control	7	21	95	NA	66(56.9)	5(4.3)	38 (32.8)	28 (24.1)	14 (12.1)	48 (41.4)	NA
Louis et al. (21)	France	Case-control	7	80	101	NA	127 (70.0)	13 (7.2)	132 (73.0)	54 (30.0)	22 (12.0)	52 (29.0)	22(12.0)
Yan et al. (22)	China	Cohort	8	115	767	71 (68,77)	440(49.9)	83(9.4)	NA	277 (31.4)	86 (9.8)	515 (58.2)	41(4.7)
Chan et al. (23)	United States	Case-control	8	1,835	2,158	64 (56,78)	2,289 (57.3)	420 (11.0)	1,527 (38.0)	1,019 (26.0)	NA	396 (10.0)	NA
Tan et al. (24)	China	Cohort	8	40	377	45.2 ±17.7	198 (47.3)	NA	55 (13.2)	19 (4.6)	16 (3.8)	26 (6.2)	6 (1.4)
Hamilton et al. (25)	England	Case-control	7	210	822	71 (56,83)	569 (55.1)	144 (14.0)	NA	134(13.0)	259 (25.1)	129 (12.5)	72 (7.0)
Lee et al. (26)	United States	Cohort	8	294	708	66 (53,76)	619 (62.0)	138 (14.0)	597 (60.0)	378 (38.0)	81 (8.0)	131 (13.0)	NA
Rubin et al. (27)	France	Cohort	8	57	14	61.2 ± 12.2	55 (77.0)	NA	43 (61.0)	21 (30.0)	8 (11.0)	17 (24.0)	NA
Xu et al. (28)	China	Cohort	7	263	408	65 (56,73)	434 (65.0)	NA	287 (43.0)	131 (20.0)	37 (6.0)	87 (13.0)	20 (3.0)
Pelayo et al. (29)	United States	Case-control	7	110	113	NA	115(51.6)	NA	180 (80.7)	104 (46.6)	27 (12.1)	59 (26.5)	NA
Li et al. (30)	China	Cohort	7	48	59	70 (64,78)	69 (64.5)	5 (4.7)	73 (68.2)	22 (20.6)	23 (21.5)	33 (30.8)	NA
Xia et al. (31)	China	Cohort	8	41	40	66.6 ± 11.4	54 (66.7)	3 (3.7)	43 (53.1)	19 (23.5)	NA	17 (21.0)	NA
Luther et al. (32)	Sweden	Case-control	6	51	6	NA	44(77.2)	NA	30 (52.6)	16 (28.1)	14 (24.6)	6 (10.5)	4(7.0)
Peng et al. (33)	China	Case-control	6	285	3,735	61 (50,69)	1,912 (47.6)	100 (2.5)	852 (21.2)	424 (10.5)	NA	270 (6.7)	NA
Wang et al. (34)	China	Case-control	7	136	139	69 (62,77)	161 (58.4)	16 (5.8)	150 (54.5)	62 (22.5)	37 (13.5)	35 (12.7)	NA
Lim et al. (35)	Korea	Case-control	8	30	130	NA	86(53.8)	NA	77 (48.1)	50 (31.3)	16 (10.0)	31 (19.4)	26(16.3)
Wang et al. (36)	China	Cohort	7	12	104	62 (55,69)	62 (53.4)	NA	47 (40.5)	20 (17.2)	3 (2.6)	12 (10.3)	10 (8.6)
Nimkar et al. (37)	United States	Case-control	8	179	148	71 (59,82)	182 (55.7)	40 (12.2)	209 (63.9)	139 (42.5)	44 (13.5)	98 (29.9)	66(20.2)
Hectors et al. (38)	United States	Case-control	7	16	29	65 (24,97)	23 (51.0)	NA	26 (57.8)	13 (28.9)	NA	11 (24.4)	NA
Ng et al. (39)	United States	Cohort	8	3,854	5,803	NA	5,747 (59.5)	492 (5.1)	5,730 (59.3)	3,469 (35.9)	610 (6.3)	2,040 (21.1)	754(7.8)
Fominskiy et al. (40)	Italy	Case-control	8	72	24	NA	80 (83.3)	6 (6.3)	42 (43.8)	16 (16.7)	7 (7.3)	13 (13.5)	3(3.1)
Hansrivijit et al. (41)	United States	Cohort	8	115	168	64.1 ± 15.9	159 (56.2)	66 (23.3)	189 (66.8)	108 (38.2)	73 (25.8)	53 (18.7)	NA
Paek et al. (42)	Korea	Case-control	6	28	676	NA	210 (29.8)	NA	226 (32.1)	123 (17.5)	NA	NA	NA
Chaibi et al. (43)	European	Case-control	7	55	156	60.0 ± 11.0	163 (77.0)	18 (8.0)	107 (51.0)	78 (37.0)	NA	28 (13.0)	NA
Sang et al. (44)	China	Case-control	8	92	118	64 (56,71)	131 (62.4)	10 (4.8)	98 (46.7)	44 (21.0)	5 (2.4)	23 (11.0)	14 (6.7)
Cheng et al. (45)	China	Cohort	8	99	1239	63 (50,71)	711 (51.0)	21 (2.0)	499 (36.0)	241 (17.0)	77 (6.0)	NA	62 (5.0)
Lin et al. (46)	China	Case-control	6	6	27	59.9 ± 12.8	23 (69.7)	3 (9.1)	15 (45.5)	6 (18.2)	NA	2 (6.1)	3 (9.1)
Zhang et al. (47)	China	Cohort	7	37	357	56 (42,67)	186 (47.2)	NA	115 (29.2)	47 (11.9)	23 (5.8)	38 (9.6)	24 (6.1)

*Data are presented as the means ± SD, n (%) or median (interquartile range). COPD, chronic obstructive pulmonary disease; CVD, cardiovascular diseases; CKD, chronic kidney disease; NA, data not available*.

### Analysis of Risk Factors for AKI in COVID-19 Patients

#### General Risk Factors

**Figure 2:**
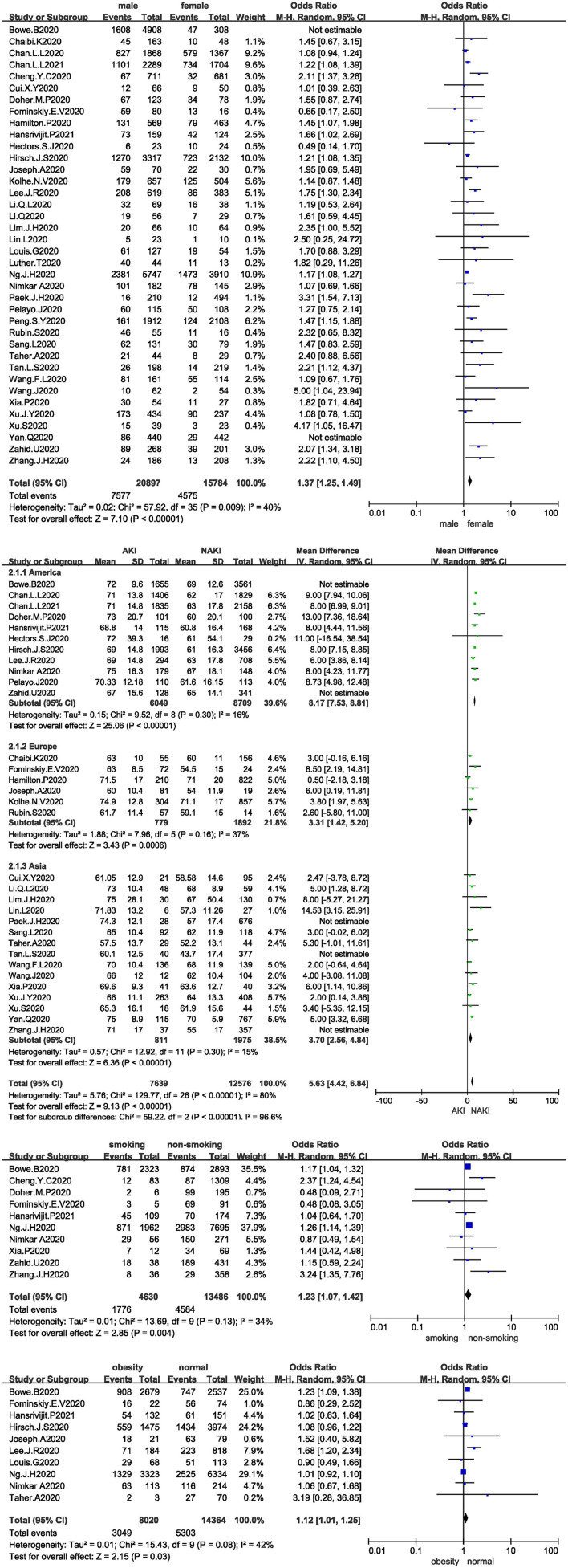
**(A)** Forest plot showing the relationship between sex and AKI in COVID-19 patients. **(B)** Forest plot showing the relationship between age and AKI in COVID-19 patients. **(C)** Forest plot showing the relationship between smoking and AKI in COVID-19 patients. **(D)** Forest plot showing the relationship between obesity and AKI in COVID-19 patients.

All studies analysed the relationship between sex and the development of AKI in COVID-19 patients, and 36 studies were included after the sensitivity analysis. The I^2^ test showed I^2^ = 40%, indicating that no heterogeneity existed among the studies. The random-effects model was used to pool the data, yielding an OR of 1.37 (95% CI 1.25–1.49, Z = 7.1, *P* < 0.00001), suggesting that male sex is a risk factor for AKI (Figure 2A).

Thirty-two studies analysed the relationship between age and the development of AKI in COVID-19 patients, and 27 studies were included after the sensitivity analysis. The I^2^ test showed I^2^ = 80%, indicating that a high degree of heterogeneity existed among the studies. Therefore, the studies were analysed in subgroups stratified by region. The I^2^ statistic was <50% in all subgroups. The random-effects model was used to pool the data (Figure 2B). The MD was 8.17 in the Americas (95% CI 7.53–8.81, Z = 25.06, *P* < 0.00001), 3.31 in Europe (95% CI 1.42–5.2, Z = 3.43, *P* = 0.0006), and 3.7 in Asia (95% CI 2.56–4.84, Z = 6.36, *P* < 0.00001), suggesting that age is a risk factor for AKI; the older the patient is, the higher the risk of AKI.

Only 10 studies analysed the relationship between smoking and the development of AKI in COVID-19 patients. The I^2^ test showed I^2^ = 34%, indicating that no heterogeneity existed among the studies. The random-effects model was used to pool the data, yielding an OR of 1.23 (95% CI 1.07–1.42, Z = 2.85, *P* = 0.004), suggesting that smoking is a risk factor for AKI (Figure 2C).

Ten studies analysed the relationship between obesity and the development of AKI in COVID-19 patients. The I^2^ test showed I^2^ = 42%, indicating slight heterogeneity among the studies. The random-effects model was used to pool the data, yielding an OR of 1.12 (95% CI 1.01–1.25, Z = 2.15, *P* = 0.03), suggesting that obesity is a risk factor for AKI (Figure 2D).

#### Comorbidities

**Figure 3:**
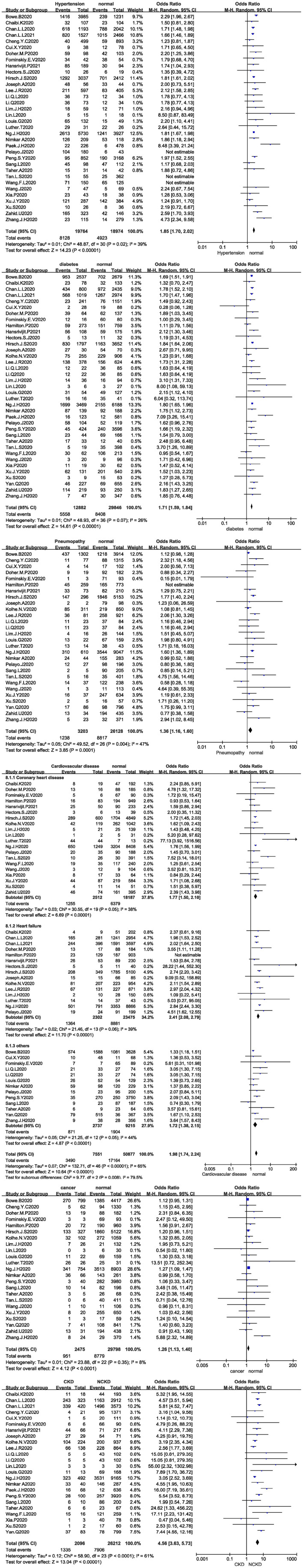
**(A)** Forest plot showing the relationship between hypertension and AKI in COVID-19 patients. **(B)** Forest plot showing the relationship between diabetes and AKI in COVID-19 patients. **(C)** Forest plot showing the relationship between pneumopathy and AKI in COVID-19 patients. **(D)** Forest plot showing the relationship between cardiovascular disease and AKI in COVID-19 patients. **(E)** Forest plot showing the relationship between cancer and AKI in COVID-19 patients. **(F)** Forest plot showing the relationship between CKD and AKI in COVID-19 patients.

Thirty-four studies analysed the relationship between hypertension and the development of AKI in COVID-19 patients, and 31 studies were included after the sensitivity analysis. The I^2^ test showed I^2^ = 39%, indicating that no heterogeneity existed among the studies. The random-effects model was used to pool the data, yielding an OR of 1.85 (95% CI 1.70–2.02, Z = 14.23, *P* < 0.00001), suggesting that hypertension is a risk factor for AKI (Figure 3A).

Thirty-seven studies analysed the relationship between diabetes and the development of AKI in COVID-19 patients. The I^2^ test showed I^2^ = 26%, indicating that no heterogeneity existed among the studies. The random-effects model was used to pool the data, yielding an OR of 1.71 (95% CI 1.59–1.84, Z = 14.61, *P* < 0.00001), suggesting that diabetes is a risk factor for AKI (Figure 3B). Twenty-eight studies analysed the relationship between pneumopathy and the development of AKI in COVID-19 patients, and 27 studies were included after the sensitivity analysis. The I^2^ test showed I^2^ = 47%, indicating that slight heterogeneity existed among the studies. The random-effects model was used to pool the data, yielding an OR of 1.36 (95% CI 1.16–1.6), Z = 3.85, *P* = 0.00001), suggesting that pneumopathy is a risk factor for AKI (Figure 3C).

Thirty-five studies analysed the relationship between cardiovascular disease and the development of AKI in COVID-19 patients. The I^2^ test showed I^2^ = 65%, indicating that a high degree of heterogeneity existed among the studies. Therefore, the studies were analysed in subgroups stratified by study type. I^2^ was <50% in all subgroups. The random-effects model was used to pool the data (Figure 3D). Coronary heart disease was associated with an OR of 1.77 (95% CI 1.50–2.10, Z = 6.69, *P* < 0.00001), and heart failure was associated with an OR of 2.41 (95% CI 2.08–2.79, Z = 11.7, *P* < 0.00001). Other cardiovascular disease, including other types of heart disease and types not described were associated with an OR of 1.72 (95% CI 1.38–2.15, Z = 4.87, *P* < 0.00001). The results suggested that cardiovascular disease is a risk factor for AKI.

Twenty-three studies analysed the relationship between cancer and the development of AKI in COVID-19 patients. The I^2^ test showed I^2^ = 8%, indicating that no heterogeneity existed among the studies. The random-effects model was used to pool the data, yielding an OR of 1.26 (95% CI 1.13–1.40), Z = 4.12, *P* < 0.00001), suggesting that cancer is a risk factor for AKI (Figure 3E).

Twenty-four studies analysed the relationship between CKD and the development of AKI in COVID-19 patients. The I^2^ test showed I^2^ = 61%, indicating that a high degree of heterogeneity existed among the studies. The random-effects model was used to pool the data, yielding an OR of 4.56 (95% CI 3.63–5.73, Z = 13.04, *P* < 0.00001), suggesting that CKD is a risk factor for AKI (Figure 3F).

#### Supportive Treatment

**Figure 4:**
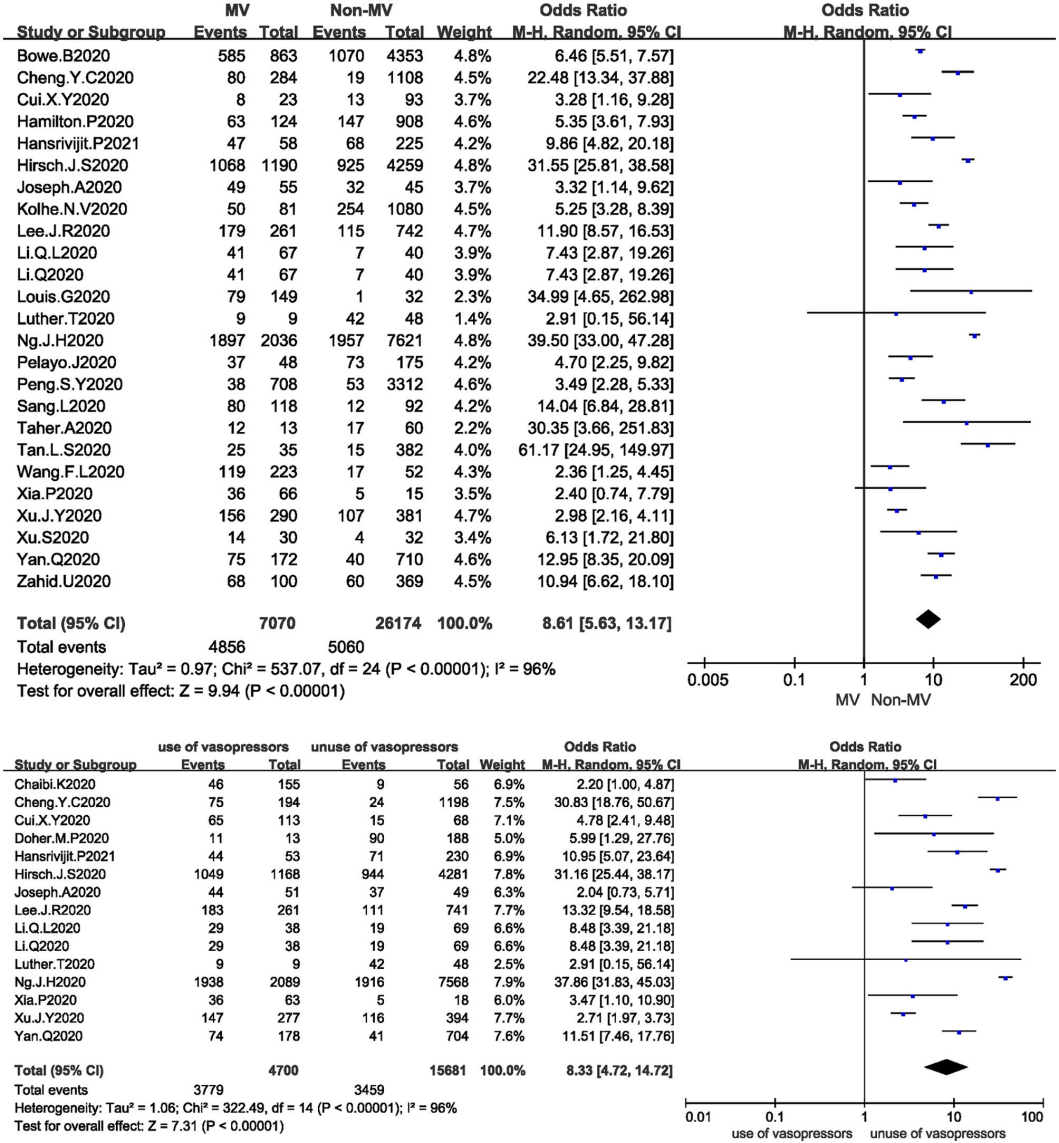
**(A)** Forest plot showing the relationship between mechanical ventilation and AKI in COVID-19 patients. **(B)** Forest plot showing the relationship between the use of vasopressors and AKI in COVID-19 patients.

Twenty-five studies analysed the relationship between mechanical ventilation and the development of AKI in COVID-19 patients. The I^2^ test showed I^2^ = 96%, indicating that a high degree of heterogeneity existed among the studies. A sensitivity analysis and subgroup analysis were performed, but the heterogeneity could not be reduced. The random-effects model was used to pool the data, yielding an OR of 8.61 (95% CI 5.63–13.17, Z = 9.94, *P* < 0.00001), suggesting that mechanical ventilation is a risk factor for AKI (Figure 4A).

Fifteen studies analysed the relationship between the use of vasopressors and the development of AKI in COVID-19 patients. The I^2^ test showed I^2^ = 96%, indicating that a high degree of heterogeneity existed among the studies. The random-effects model was used to pool the data, yielding an OR of 8.33 (95% CI 4.72–14.72), Z = 7.31, *P* < 0.00001), suggesting that the use of vasopressors is a risk factor for AKI (Figure 4B).

#### Bias Assessment

**Figure 5:**
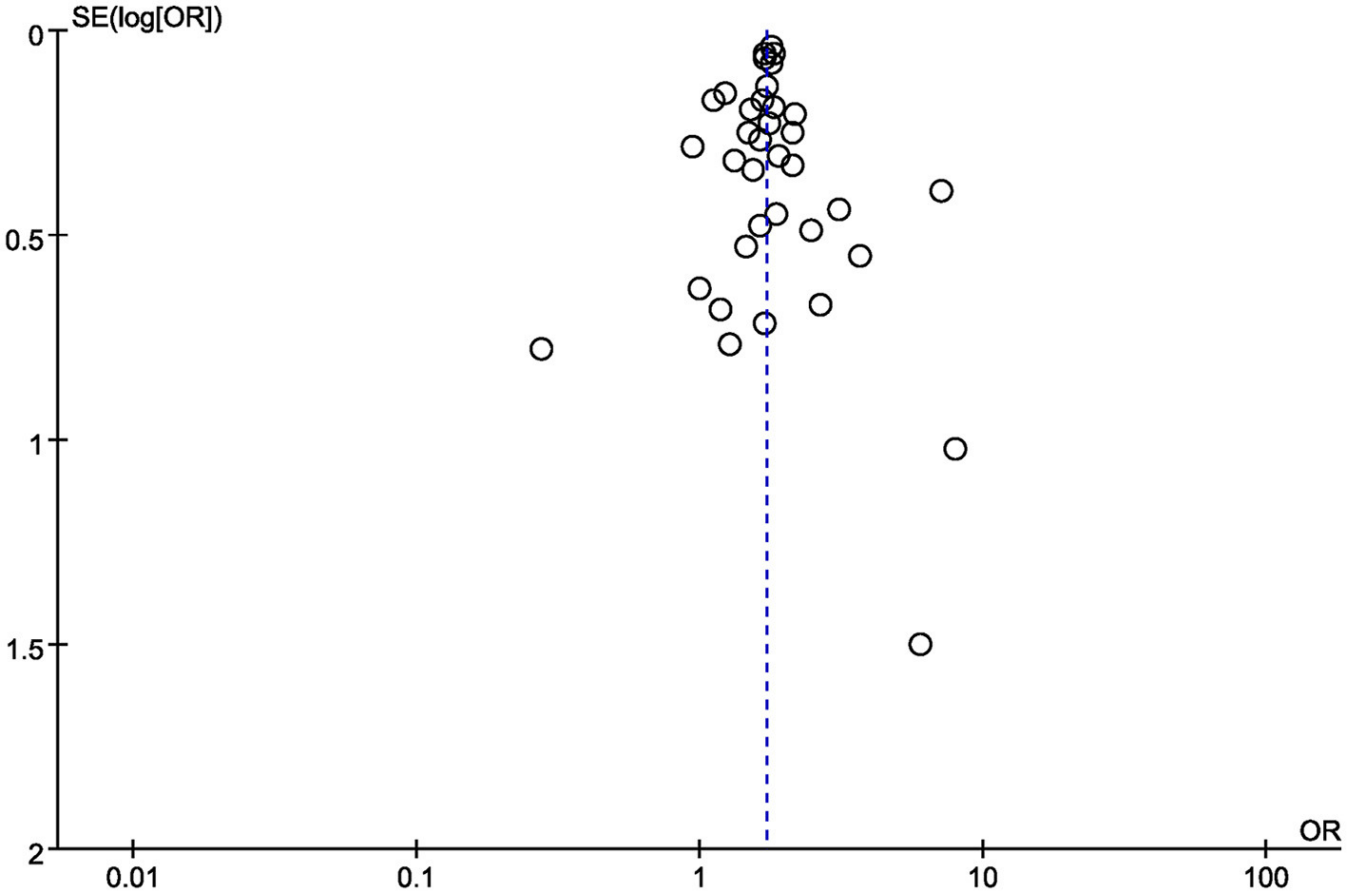
Funnel plot for the relationship between diabetes and the development of AKI in COVID-19 patients.

Finally, funnel plots were constructed to qualitatively analyse the publication bias among the included studies. The relationship between diabetes and AKI in COVID-19 patients was used as an example. The funnel plots displayed symmetrical distributions, with no obvious publication bias (Figure 5).

## Discussion

Our study included 42,779 subjects in 38 studies and explored the risk factors for AKI in adult patients with COVID-19. To our knowledge, this study had the largest number of included studies and the largest sample size. Although research has investigated the clinical characteristics, pathobiology, treatment methods and other related factors, means of improving the prognosis of AKI remain to be identified, and further research is needed to reduce the adverse consequences for patients. Recently, Fisher et al. from New York reported higher rates of AKI in those with COVID-19 than in those who tested negative for this disease (48). Currently, the mechanism underlying kidney injury in patients with COVID-19 is believed to involve SARS-CoV-2 directly attacking intrinsic renal cells. SARS-CoV-2 is a cytopathic virus that passes through the membrane protein ACE2 to enter host cells (49). The expression level of ACE2 in renal cells ranks 4th among the 55 tissue types and 6 blood cell types, with consistent standardised expression levels. Therefore, patients with COVID-19 have a relatively higher risk of developing AKI. Kidney histology in patients with COVID-19 has shown the presence of acute tubular necrosis, moderate-to-severe lymphocytic infiltration and collapsing glomerulopathy (50). Invasion by SARS-CoV-2 causes the T lymphocyte count to decrease, especially CD4+T cells and CD8+T cells, and the levels of IL-6, IL-10, IL-2, and interferon to increase (51). These inflammatory cytokine levels are increased due to the recruitment and infiltration of inflammatory cells and participate in tissue damage and repair, resulting in cell, tissue and organ oedema and other injuries. SARS-CoV-2 can penetrate the proximal tubule by connecting ACE2 to CD147 and can also penetrate podocytes by linking ACE2 (52). Viruses can cause podocyte dysfunction, resulting in glomerular disease. SARS-CoV-2 results in an imbalance in renin-angiotensin system (RAS) activation and promotes the progression of glomerular dysfunction, fibrosis, vasoconstriction, and inflammation (53). Infection with SARS-CoV-2 can also activate the coagulation system, leading to renal vascular injury (54). AKI is considered a negative prognostic factor with regard to survival (55). Mortality was found to be significantly more common in patients with hospital-acquired AKI and patients with intrinsic AKI. Identifying the risk factors for AKI in these patients may help reduce mortality due to COVID-19.

Our study found that male sex, age, smoking, obesity, hypertension, diabetes, pneumopathy, cardiovascular disease, cancer, CKD, mechanical ventilation and use of vasopressors were independent risk factors for AKI in adult patients with COVID-19. Previous studies have confirmed increased severity of and mortality due to COVID-19 in elderly patients (56). A recent study comparing the clinical characteristics and results in COVID-19 patients of different ages showed that the symptoms in elderly patients were more atypical, and these patients had more comorbidities, secondary infections, organ injuries, immunodeficiencies and critical illness (57). Many comorbidities in the elderly population, such as hypertension, diabetes and CKD, are treated with ACE inhibitors (ACEIs) and angiotensin II receptor blockers (ARBs), which upregulate ACE2, thereby increasing the risks of SARS-CoV-2 infection and severe disease. The proliferative ability of stem cells, which play an important role in renal cell repair, gradually decreases with age (58). Our study showed that age was an independent risk factor for AKI; the older the patient was, the higher the risk of AKI. Another study (59) showed that advanced age was an independent risk factor for AKI, which was consistent with our conclusion.

Recently, chronic kidney disease (CKD) emerged as the most common risk factor for severe COVID-19, and alarmingly, after age, it is also the strongest risk factor for severe COVID-19 (60). The removal of CKD as a risk factor would decrease the percentage of the global population at increased risk of severe COVID-19 from 22 to 17% (61). Thus, CKD explains the increased risk of severe COVID-19 in approximately one in four individuals at high risk worldwide, which is equivalent to 5% of the global population or 86,530,000 persons. CKD has emerged not only as the most prevalent comorbidity that is associated with an increased risk for severe COVID-19 but also as the comorbidity that conveys the highest risk for severe COVID-19. The increased risk is evident even below the threshold for the estimated glomerular filtration rate (eGFR) that is used to define CKD, and the risk increases as the eGFR decreases, with the highest risk in patients on renal replacement therapy. Some research has demonstrated that patients with CKD, particularly those with end-stage kidney disease (ESKD), have immune dysregulation and increased susceptibility to infections (62). For many patients with CKD, renin-angiotensin-aldosterone system (RAAS) blockade is a mainstay of treatment. The potential detrimental effect of the ongoing use of ACEIs or ARBs is the upregulation of ACE2, which could increase the ability of the virus to enter the cells.

Some studies have shown that the protein expression level of ACE2 in smokers is significantly higher than that in non-smokers. This may be evidence that a history of smoking is a risk factor for AKI in patients with COVID-19. We also found that hypertension, diabetes and cardiovascular disease were independent risk factors for AKI, and these comorbidities were associated with micro- and macrovascular complications, all of which affected the renal blood flow. Any minor haemodynamic or nephrotoxic insult can lead to substantial AKI in these patients. Due to the use of ACEIs, the expression of the ACE2 protein in hypertensive patients is higher than that in non-hypertensive patients (63). This increases the risk of COVID-19-associated AKI by increasing the sites for virus binding. Patients with underlying cardiovascular disease and hypertension have been reported to have significantly high-case fatality rates compared with patients without these underlying comorbidities (10.5 and 6% mortality, respectively, compared with 0.9% mortality without underlying comorbidities) (64). Our study have stated that diabetes and obesity as risk factors for AKI in Covid-19 patients, therefore controlling those factors with anti-diabetic drugs may help in reducing the burden from the disease and mortality rate. Several anti-diabetic drugs have shown beneficial and neutral effects towards Covid-19. Some preliminary data from retrospective studies have confirmed a reduction in death rates in metformin users compared with non-users in patients with T2DM hospitalised for COVID-19 (65). Patients with diabetes should be advised to continue taking metformin drugs despite COVID-19 infection status (66). One meta-analysis suggests that pre-admission use of GLP-1RA may offer beneficial effects on Covid-19 mortality in patients with diabetes mellitus (67).

One study showed that coronavirus pneumonia brought about a 24% mortality in individuals with cancer while a 3% mortality was observed with non-cancer patients (68). Related studies have reported that AKI is a common complication in patients with malignant tumours, and the incidence of AKI in such patients is as high as 30%, which may be attributed to the renal toxicity of anti-cancer regimens (69). The incidence of AKI is markedly elevated in mechanically ventilated patients. Our study also confirms that mechanical ventilation is a risk factor for AKI in adult patients with COVID-19. Mechanical ventilation can increase the pressure in the thoracic cavity, resulting in reduced venous return, decreased cardiac output and decreased renal perfusion. In addition, mechanical ventilation can also induce pro-inflammatory reactions, change the neuro-humoural system, affect glomerular filtration, and cause or promote the occurrence and development of AKI (70).

## Conclusions

AKI is a common and serious complication of COVID-19. Our meta-analysis indicates that male sex, age, smoking, obesity, hypertension, diabetes, pneumopathy, cardiovascular disease, cancer, CKD, mechanical ventilation and the use of vasopressors were independent risk factors for AKI in adult patients with COVID-19. Clinicians need to be aware of these risk factors to reduce the incidence of AKI. Some anti-diabetic drugs may help in reducing the burden from the disease and mortality rate. Several anti-diabetic drugs have shown beneficial and neutral effects towards Covid-19, such as metformin, GLP-1RA, and DPP-4 inhibitor (65, 67, 71). If we use metformin, GLP-1RA as the drug of choice for the management of patients with type 2 diabetes during the COVID-19 pandemic, it may improve patient outcomes, especially those with cardiovascular risk factors.

The limitations of this study are as follows: there were differences in the ethnicities of the subjects, numbers of cases, research methodologies and regions among the studies included in this analysis, and there was heterogeneity after the combination of some risk factors. The sample sizes of the included patients were not large enough for some factors. Further studies are still needed.

Although we identified 11 risk factors for AKI, we believe that there are more potential risk factors for AKI that need to be investigated in future studies. Early identification and early intervention can reduce the occurrence of AKI and further improve the prognosis of patients with COVID-19.

## Data Availability Statement

The original contributions presented in the study are included in the article/supplementary material, further inquiries can be directed to the corresponding author/s.

## Author Contributions

XC: statistical analysis. XC and GW: literature research and selection, data extraction. XC, GW, and JZ: data analysis/interpretation. LY: funds collection, conception and design of the study, and revised manuscript. All authors interpreted the results and contributed to critical review of the manuscript.

## Conflict of Interest

The authors declare that the research was conducted in the absence of any commercial or financial relationships that could be construed as a potential conflict of interest.

## Publisher's Note

All claims expressed in this article are solely those of the authors and do not necessarily represent those of their affiliated organizations, or those of the publisher, the editors and the reviewers. Any product that may be evaluated in this article, or claim that may be made by its manufacturer, is not guaranteed or endorsed by the publisher.
